# Fabrication of Polymer Optical Fibre (POF) Gratings

**DOI:** 10.3390/s17030511

**Published:** 2017-03-04

**Authors:** Yanhua Luo, Binbin Yan, Qijin Zhang, Gang-Ding Peng, Jianxiang Wen, Jianzhong Zhang

**Affiliations:** 1 Photonics & Optical Communications, School of Electrical Engineering and Telecommunications, University of New South Wales, Sydney, NSW 2052, Australia; yanhua.luo1@unsw.edu.au; 2 State Key Laboratory for Modification of Chemical Fibers and Polymer Materials, Donghua University, Shanghai 201600, China; 3 State Key Laboratory of Information Photonics and Optical Communications, Beijing University of Posts and Telecommunications, Beijing 100876, China; yanbinbin@bupt.edu.cn; 4 CAS Key Laboratory of Soft Matter Chemistry, Department of Polymer Science and Engineering, University of Science and Technology of China, Hefei 230026, China; zqjm@ustc.edu.cn; 5 Key Laboratory of Specialty Fiber Optics and Optical Access Networks, Shanghai University, Shanghai 200072, China; wenjx@shu.edu.cn; 6 Key Lab of In-fiber Integrated Optics, Ministry of Education, Harbin Engineering University, Harbin 150001, China; zhangjianzhong@hrbeu.edu.cn

**Keywords:** polymer optical fibre (POF), POF gratings, photosensitivity, grating fabrication, phase mask, point-by-point, sensor

## Abstract

Gratings inscribed in polymer optical fibre (POF) have attracted remarkable interest for many potential applications due to their distinctive properties. This paper overviews the current state of fabrication of POF gratings since their first demonstration in 1999. In particular we summarize and discuss POF materials, POF photosensitivity, techniques and issues of fabricating POF gratings, as well as various types of POF gratings.

## 1. Introduction

With the continuing development of material and fabrication technologies over the last three decades, the transmission attenuation of POFs has been greatly decreased. POFs are advantageous for home networks as well as storage interconnections [1]. These developments have been well described in review articles [1,2,3,4,5,6,7]. There are also some review papers covering conventional POFs and doped POFs for communications [8,9]. Besides conventional POFs, material properties, fabrication and applications of microstructured POFs (mPOFs) have also been reviewed [10,11,12,13]. In general POF has several distinctive advantages over silica fibre for sensing applications [14]. POF sensors have been discussed as a special class of fibre optic sensors included in fibre sensor reviews by Bartlett [15], Grattan [15] and Zubia [3]. Recently reviews specific on POF sensors have been presented by Peters [16], Bilro [17], and Granville [18]. Thematic reviews on the smart textiles, structural health monitoring (SHM), and aircraft SHM applications of POF sensors have also been described by Zhang [19], Kuang [20] and Zubia [21], respectively. Among all different types of POF sensors, those based on fibre gratings written in POFs have attracted great attention since they were first demonstrated in 1999, due to their quite distinctive properties such as higher sensitivity and larger dynamic range compared to their silica counterparts [22]. A number of earlier reviews on POF gratings and their sensing applications had appeared in reports by Peng [23], Canning [24], Kuang [20], Argyros [12,13], Peters [16], Bilro [17], Zubia [21], Granville [18] and Farrell [25]. The difficulties and challenges encountered during the photo-inscription of gratings in mPOFs have also been reviewed by Berghmans [26]. Recently, more specific reviews on POF grating-based optical sensors have been given by Webb [27,28]. Webb summarized POF gratings’ properties, photosensitivity, sensitivities and their sensing applications. New advances in POF Bragg gratings regarding the produce of smooth POFs end faces with high quality and the increase of quality in the production of FBGs have also been summarized by Bilro [29]. These previous reviews regarding POF gratings are mainly focused on the properties of POF gratings as well as their sensing applications. However, there hasn’t been any systematical review on the fabrication of POF gratings. Through the intense work of nearly two decades, a variety of POF grating techniques and applications have been developed. Therefore, it will be useful to summarize the work conducted on POF gratings during the last 18 years. The review of their fabrication will be appreciated by the community and is also a good starting point for researchers who are new to the field. This paper discusses published work, work being performed at present, as well as prospective future work in POF gratings and their applications. In Section 2, we briefly introduce the general aspects of POF gratings, usual POF materials and their photosensitivity and photosensitive POFs. In Section 3, we describe and discuss techniques and issues of fabricating POF gratings. Different types of POF gratings developed so far have been summarized in Section 4.

## 2. POF Gratings

Optical fibre Bragg gratings (FBGs) have a periodic (or quasi-periodic) modulation of the refractive index along the fibre core. The FBG preferentially reflects light with a wavelength, **λ_B_**, which is determined by the Bragg condition [30]:

**λ_B_** = 2n_eff_**Λ** (1)

where n_eff_ is the effective index of optically guided mode and Λ is the period of the modulation. For sensing applications, gratings are very useful because any change of strain or temperature applied to them changes both the period and index of the grating, resulting in a shift of **λ_B_**. The periodic structure is normally produced by exploiting the intrinsic sensitivity of the core material to inscription light (λ_w_) and exposing the fibre to a periodic intensity pattern produced by interfering two light beams. The amplitude of index modulation is very closely related to the photosensitivity of POF materials. Thus a proper writing wavelength is to be selected according to the photosensitivity of materials. In the regard we will discuss POF materials and their photosensitivity, photosensitive POFs, as well as inscription and operating wavelength of POF gratings in the following subsections.

### 2.1. POF Materials And Their Photosensitivity

The material information and photosensitivity of various reported POFs have been summarized and listed in Table S1. Poly(methyl methacrylate) (PMMA), polycarbonates (PC), polystyrene (PS), cyclic olefin copolymer (COC produced by TOPAS, Frankfurt, Germany) and amorphous fluoropolymer (CYTOP produced by Asahi Glass, Tokyo, Japan) are the most popular optical polymers used for the POF fabrication. Their chemical structures are listed in Table 1. The material properties, like refractive index (**n**), glass transition temperature (**T_g_**) and melting temperature (**T_m_**), thermal expansion coefficient (α), thermo-optic coefficient (**dn/dT**), stress-optic coefficient and moisture absorption are also listed in Table 1. The **n** will determine **λ_B_** when **Λ** is fixed. **T_g_** and **T_m_** will determine both the processing and maximum operation temperatures.

As seen from Table 1, PC is superior to PMMA in mechanical properties and has a higher operation temperature thanks to its higher T_g_. Although its polymerization and modification is not as easy as that of PMMA, Fasano et al. have started the fabrication of PC mPOF grating strain sensors for high temperature resistant [44]. PS, without distinct advantages over PMMA, is rarely used to fabricate photosensitive POFs. The phenyl groups make it especially vulnerable to ultraviolet (UV) light. The reported photosensitive POF used for POF gratings only contains 5% PS [45,46,47]. CYTOP has the lowest transmission loss among them [16].

The reported CYTOP POF gratings are based on the commercial multimode (MM) graded-index (GI) POF [48,49], although SM POF with CYTOP core has recently been drawn through a preform by further polymerization of MMA around MM GI POF by Zhou et al. [50]. COC is an amorphous transparent copolymer, which has already been used for the fabrication of POF for their attractive feature of low moisture absorption (0.01%) and good chemical resistance though its **T_g_** is lower than the typical PMMA [31,51,52,53,54].

PMMA is still the most common and popular material [31], although various materials have been tested and used for the fabrication of POF gratings as listed in Table S1. The reason is that it can be readily tailored to meet varying requirements, such as the refractive index, drawing temperature, etc. by the co-polymerization of methyl methacrylate (MMA) with other acrylic monomers (e.g., ethyl methacrylate (EMA), benzyl methacrylate (BzMA), etc.) or the incorporation of small molecules (e.g., diphenyl sulfide (DPS)) [38,45,47,55,56].

For optical inscription of POF gratings, the photosensitivity of the material is very important. Until now, there still exist various understandings with regard to photosensitivity as well as precise mechanism(s) of the inscription in PMMA material [28]. Research on the photosensitivity could be traced back to the early 1970s when it was done on bulk PMMA at Bell Labs. In the following, Tomlinson et al. discovered that properly prepared PMMA bulk exhibited a substantial increase in refractive index of 10^−3^ after irradiation of 325 nm light accompanied by the density increase, where they attributed it to the possible photocrosslinking [57]. The photopolymerization of residual monomers has also been found in the formation of gratings in pure PMMA by a mercury lamp, but it will have a refractive index decrease prior to a final increase of 10^−2^ [58]. The initial refractive index decrease may be attributed to the photodegradation of PMMA under UV radiation [59], which will generate monomers and radical groups for later chemical reactions such as the photopolymerization and photocrosslinking. Further evidence of the competitive co-existence of photodegradation and photopolymerization is provided by the recent work of Saez-Rodriguez et al. [60]. The evidence of photodegradation comes from the strong correlation between strain applied on gratings inscription and reflectivity, where the stress significantly enhanced the photodegradation [61], while the measured refractive index change in the fiber is positive, which confirm the existence of further polymerization.

Generally, the photosensitivity of usual POF materials such as PMMA is low. The low photosensitivity will bring a series of drawbacks, like poor grating performance, short writing light wavelength and long FBG fabrication time, resulting in the optical damage, low mechanical strength and poor long term stability of the POF gratings [62,63]. Therefore, functional dopants, such as fluorescein, *trans*-4-stilbenemethanol (TSB), benzildimethylketal (BDK), 9-vinylanthracene (VA), etc. have been introduced into POF for the enhancement of the photosensitivity, as listed in Table S1 [22,64,65,66,67,68,69]. The incorporation can be either through the simple doping with small molecules [22,64,65], or through the co-polymerization with photosensitive monomers [66,67,69]. The photosensitivity of the latter introduction can be more stable. In addition, the photosensitivity can also be enhanced by the straining during the grating inscription [61] or the etching of photosensitive POF cladding [47,70]. As shown in Table S1, the change of the refractive index by radiation is ranged from 10^−5^ to 10^−3^. There are several reasons resulted in the large variation of the values: 1) non-uniform test conditions; 2) the change of refractive index in POF gratings often calculated from the reflectivity, which is much dependent upon inscription conditions, grating length, etc.; 3) the difference of host materials, dopant concentration, etc.

The photosensitivity of POF gratings can be classified into two types: reversible and irreversible. The photosensitivity of two dopants, azobenzene [67,69,71,72,73,74] and Photosol 7-049 [56] is found to be reversible. The former can be photoaligned under linearly polarized light and erased by circularly polarized light, which will write/erase the photoinduced birefringence for forming/erasing of fibre gratings. The latter dopant can alternate between two isomeric forms by a photoinduced ring opening reaction during which refractive index will be changed. However, there are many materials being the irreversible. Their photosensitivity could be linked to photoisomerization of TSB [64], photodegradation of polymers co-polymerized with MMA and methyl vinyl ketone (MVK) [75], photocrosslinking based on photo-addition of anthracene groups [66,76], photopolymerization of residual monomers [77], photo-modification by ultrashort pulse laser [78,79,80,81,82]. So far, most POF gratings for photonic applications have been made of materials with irreversible photosensitivity.

### 2.2. Photosensitive POF

Generally, single mode (SM) POF is more preferred for POF gratings as it is suitable for device applications, such as fibre lasers and amplifiers, optical switches and modulators, broadband tunable FBGs, etc. [83] though there have been many reports regarding MM POF gratings [22,64,65,66,76,84,85,86,87,88,89,90,91,92]. For SM transmission of step index (SI) POF, the normalized frequency should obey the following equation [93]:

(2) $$V=\frac{2\pi }{\lambda }\cdot a\sqrt{n_{\mathrm{co}}^{2}-n_{\mathrm{cl}}^{2}}<2.4$$

where 2*a* and *λ* are the diameter of fibre core and the operating wavelength, respectively. The refractive index of the core (n_co_) and cladding (n_cl_) will mainly rely on the materials and material compositions, estimated with parameters in Table 1 using the Lorentz-Lorenz equation [55]. In addition, SM POFs have advantages like: low temperature fabrication, compatibility with functional organics, flexible synthesis and easy mechanical processing. The first SM SI POF was fabricated by Kuzyk et al. in 1991 with high loss of 200 dB/km at 652 nm [94].

So far, most of step-index (SI) POFs are mainly PMMA-based ones made by bulk polymerization, as shown in Table S1. The bulk polymerization often gives more flexibility for the modification of the material properties through doping to improve the photosensitivity [22,64,65,66,67,68,69] as well as other functionalities. MM graded-index (GI) perfluorinated POF (CYTOP) is manufactured by Chromis Fiberoptics, Inc. (Warren, NJ, USA) through extrusion [48,92]. Recently, COC SI POF has also been fabricated by Woyessa et al., through the tube-in-rod method with injection molded preforms [95]. Besides the traditional SI POF, mPOF made by drilling, stacking or casting/molding have also aroused considerable interest due to the novel features like their “endlessly single-mode” properties, easy dispersion tailoring and polarization controlling, the possibility of large core SM fibre, and even the ability to make band-gap fibres [12,96].

Photosensitive POFs have mainly been reported by several research groups, listed in Table 2 [22,64,65,66,67,68,75,77,97]. These POFs have varying specifications (loss concentricity, diameter, photosensitivity, drawing induced anisotropy) [98].

### 2.3. Inscription and Operating Wavelength of POF Gratings

Photosensitivity is highly dependent upon the inscription wavelength in respect to wavelength-dependent light-matter interaction processes. The inscription wavelength, source and its related mechanism used for POF gratings fabrication are summarized and listed in Table 3. The possible processes behind photosensitivity in POF include photodegradation, photopolymerization, photocrosslinking, photoblation, optical ring cleavage, etc. Seen from Table 3, the inscription wavelength of POF gratings fabrication ranged from deep UV 248 nm to near infrared band (NIR) 800 nm. So far, the writing light sources used to create POF gratings included KrF or XeCl excimer lasers, different 325 nm UV lasers, fs lasers with different output wavelengths, Nd:YAG or Nd:YVO_4_ lasers, Ar^+^ lasers, He-Cd laser at 442 nm, or even mercury lamps with a wide UV band. However, 325 nm is the classic writing wavelength which was first reported in 1999 [22]. Currently, it is mainly coming from He-Cd laser, which are used in most of reported POF grating fabrications.

**Figure 1:**
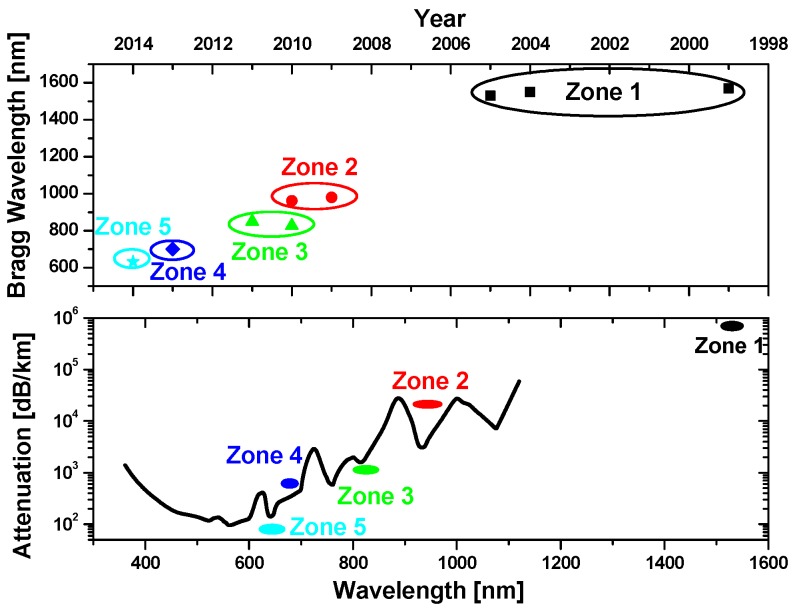
Historical evolution of the operating wavelength of PMMA POF Bragg grating since 1999 as well as the typical attenuation spectrum of PMMA POF.

Another important parameter of the POF grating is the operating wavelength (Bragg wavelength for FBGs and resonance wavelength for long-period gratings (LPGs)). The historical evolution of the operating wavelength of PMMA POF grating is plotted in Figure 1. Since the first demonstration of POF grating operated at 1570 nm in 1999 [22], most of the Bragg gratings in POFs have still been operated in the 1550 nm region due to the compatibility with existing silica FBG systems. However, compared with the typical attenuation spectrum of PMMA POF, ~1550 nm (zone 1) is indeed not the most desirable due to the high loss. In contrast, shorter wavelengths, i.e., at 600–700 nm (zone 5) with the lowest losses are more preferable as shown in Figure 1.

Therefore, a few research groups have started the fabrication of POF grating operating at wavelengths in visible or NIR regions [46,86,91,120,141]. One key recent advance is to succeed moving it from NIR band (zone 1, especially 1550 nm) to the transmission window of POF (zone 5, ~650 nm), where operating wavelength at ~633 nm has been achieved by Bundalo et al. [120]. In addition, the longest operating wavelength of FBG in COC POF has even been extended into the THz region by Zhou et al. [99].

## 3. Techniques and Issues of Fabricating POF Gratings

### 3.1. Fabrication Techniques

Since their first fabrication in 1999, different types of POF gratings have been fabricated with different writing techniques [63,64,65,69,75,77,85,91,142]. So far, there are mainly two categories of writing techniques for the fabrication of POF gratings, which are mechanical and optical methods. For the mechanical method, Hiscocks et al. adopted a simple heat imprinting for producing stable LPGs in mPOF, which is simple and low cost but only suitable for LPG instead of Bragg grating [142]. However, most of the researchers used the optical method to inscribe POF gratings. The optical method can further be classified into four kinds:

(1) Interferometeric method [56,69,74,77,78,79,80],
(2) Phase mask method [29,64,68,85,95,100,143],
(3) Amplitude mask method [67,71,72,73,75,135,136],
(4) Direct writing method [66,76,99,109,110,138,139,140].

The interferometric method is the classic method for gratings fabrication. The first SM POF gratings were fabricated with the modified Sagnac interferometeric method by Xiong et al, where the effect of the zero-order diffraction of the phase mask (designed for silica FBG fabrication) can be overcome [77]. This method has great flexibility. But it often has the coherence requirement of the writing source for the good interference.

Phase mask method is the popular method for the gratings fabrication. In 2004, Yu et al. used it with 325 nm UV laser to inscribe POF gratings in TSB doped MM POF [64]. Then in 2005, Dobb et al. used it to demonstrate the feasibility of inscribing FBGs with a 325 nm He-Cd laser in PMMA mPOF [85]. More recent POF gratings have been fabricated by this method [29,68,95,100,143]. The length of the grating can be increased by expanding/scanning the writing laser beam or moving the phase mask and fibre [47,91,124].

Amplitude mask method has mostly been adopted for POF LPG fabrication by research group in USTC. In 2005, Li et al. used amplitude mask method to write the LPGs in MVK-doped POF with a high pressure mercury lamp [75]. Writing LPGs using amplitude masks has the lowest requirement for the coherence of the writing source, and even high pressure mercury lamps can be used.

Another alternative approach of fabricating POF gratings is to directly use the focused laser beam through point by point writing. The direct point-by-point inscription was first demonstrated in the POF core without damage using 800 nm femtosecond laser by Stecher et al. in 2009 [138]. Based on this method, LPGs in mPOFs [109,110] and VA-doped SI POFs [66,76] have been fabricated. As each point will be created by the focused laser beam, the period will be limited by the diffraction limit. Therefore, direct writing of the POF gratings are also mainly for LPGs, but with suitable design, the 4th order FBGs can be located at 1518.67 nm using this method [139,140]. In addition, FBGs operated in THz region will easily be achieved with it [99].

### 3.2. Issues in POF Grating Fabrications

In terms of grating strength and linewidth, it is necessary to improve the writing conditions of the existing grating writing techniques. This is not only dependent upon the photosensitivity of POF as discussed above, but also the writing conditions such as power, incidence angle, strain applied, exposure time and etching.

#### 3.2.1. Writing Beam Intensity

Novel behaviors of the growing and erasing of polymer FBGs under UV exposure, and regrowing after the UV exposure is off in FBGs growth disclose that polymer FBG growth is a writing-power-dependent process [106]. The results from two series of mPOF gratings fabricated in two different levels of laser power further demonstrated that grating writing time depends much upon the laser power. More intensity in the core will result in shorter writing time though the reflection of gratings is not significantly influenced [120]. However, the higher power will easily damage the POF [63]. For the direct writing with 800 nm fs laser, there exists a certain threshold of pulse energy. Above the threshold, localized damage will be induced in the fibre core, while below the threshold, refractive index modifications are induced without any damage [138]. Therefore, the power selection should be balanced, taking account of the writing time, the damage of sample as well as the strength.

#### 3.2.2. Incidence Angle

The numerical study on gratings writing in microstructure fibre has demonstrated that there exist preference incidence angles [144,145]. Through the comparison of 24 mPOF gratings made with random initial orientation towards the inscription beam, Bundalo et al. have experimentally confirmed that there is a strong preference angle, labeled ГK, over the other ones [126,127]. Angles close to ГK showed fast start of inscription, rapid inscription and stronger gratings due to the direct access of the laser light to the fibre core. They have also shown that gratings can also be obtained at almost any angle for enough scattering by the holes to the fibre core, but their quality will be lower if they are not around ГK angle.

#### 3.2.3. Strain

Through analysis of a total of 54 FBGs, it can be observed that there is a strong correlation between the maximum reflectivity and the inscription strain applied [60,122]. The results demonstrate that the reflectivity increased with the tensile stress, which further hints a concomitant rise in photosensitivity by the tensile stress [60,122].

#### 3.2.4. Writing Duration

**Figure 2:**
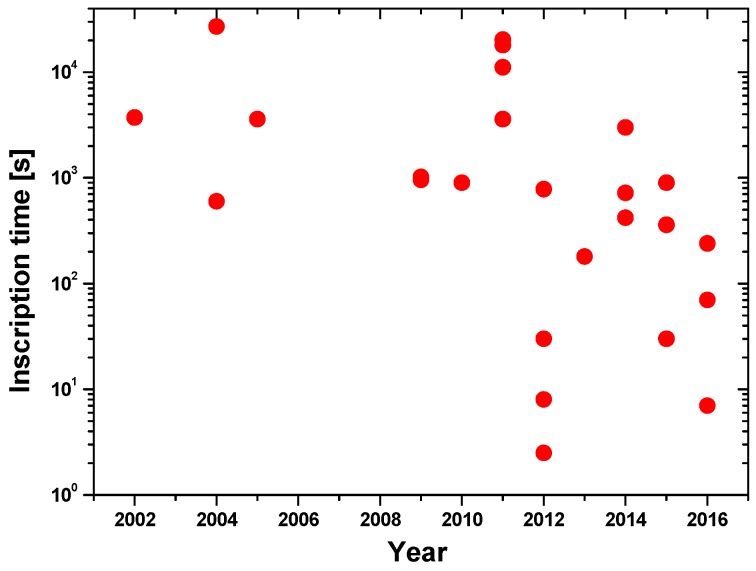
Historical evolution of inscription time of POF Bragg gratings.

The inscription of POF gratings is a time consuming process. In the early stage, it took about 1 h for PMMA POF grating to reach the maximum reflection [104]. With further UV radiation, the gratings reflection will be reduced. Although the control of the writing time is a complicated process, the reduction of the inscription time is the development trend for the improvement of writing efficiency. Figure 2 shows the historical evolution of inscription time of POF Bragg gratings [44,46,52,64,65,68,70,81,85,86,91,92,95,99,102,106,112,115,120,121,126,127,132,140].

As shown in Figure 2, the inscription reduced greatly from tens of minutes to seconds with the development of the photosensitive POF materials and inscription techniques. The direct writing technique with fs laser allows the fabrication of FBGs in mPOF in only 2.5 s [140].

#### 3.2.5. Etching

During the past few years, a great progress has been made in the development of POF gratings to achieve high grating reflectivity and sensitivity. Hu et al. [47] and Bhowmik et al. [70] have demonstrated that etching has significant enhanced reflectivity and shortened the exposure time. Meanwhile, the strain sensitivity has also been enhanced. The enhancement of the reflectivity by etching is mainly resulted from the following reasons [70]:

(1) The UV interference pattern at its core will have a higher intensity than that of an un-etched fibre, due to a strong absorption of the UV laser beam by PMMA;
(2) Due to chemical etching, penetration of the solvent will change the properties of the core, especially the photosensitivity;
(3) The fibre expansion that occurs during etching loosens the matrix of the POF material, which can allow the UV light to react more in an etched fibre than that of an un-etched fibre.

## 4. Types of POF Gratings

### 4.1. POF Bragg Gratings

Since the first fabrication of MM POF Bragg gratings in 1999 [22], many Bragg gratings have been fabricated in different POFs based on different kind of materials and mechanisms [46,49,64,77,85,103,112,120,125,141,146]. The photonic applications for optical filters in POF transmission systems and photonic sensing applications make narrow bandwidth with high reflectivity increasingly essential. In 1999, SM POF Bragg grating with a linewidth of ~0.5 nm and reflectivity of 80% at 1570 nm was fabricated [77]. In 2011, narrow bandwidths of 0.29 nm and 0.17 nm were achieved in FM mPOF and SI POF FBGs at 850 nm with 10 mm-long grating and 10 dB rejection, respectively [91]. In 2013, using a scanning technique with a short optical path, 3 dB bandwidth varied from 0.22 nm down to 0.045 nm has been achieved in long FBGs with resonance wavelengths at around 600, 850 and 1550 nm and 21 dB rejection [117,118,119]. With the development of fabrication techniques of POF gratings [44,46,52,54,64,65,68,77,81,85,91,102,103,104,105,112,117,118,119,120,121,125,128,133,143], the bandwidth has overall decreased from 1.0 nm to 0.045 nm.

### 4.2. Long Period POF Grating

Long period fibre grating (LPFG) is a type of grating with a period much larger than the wavelength and it can couple light from a guided mode into forward propagating cladding modes where it is lost due to the absorption and scattering. So far, based on different kinds of mechanisms and writing methods, many LPGs have been fabricated in SM and MM POF, SI and GI POF, as well as mPOFs as listed in Table 4 [48,67,71,72,73,75,76,78,79,82,84,87,109,110,123,135,136,138,142,147]. The point-by-point technique is the most used method used for POF LPG fabrication. As shown in Table 4, the period, operating wavelength (λ_p_) and the transmission loss dip ranged from 1 μm to 1200 μm, 510 nm to 1568 nm and 3 dB to 34 dB, respectively.

### 4.3. Special POF Gratings

#### 4.3.1. Birefringent POF Gratings

Birefringent fibre gratings have interesting properties involving the possibility to transmit or reflect different wavelengths for each polarization mode. There are several methods resulting in the birefringent fibre gratings, such as the polarized light, geometrical asymmetry in writing process and asymmetry structure in fibre [47,67,71,72,73,100,135,136]. As early as 2001, Zhang and his partners initiated the fabrication of long period birefringent gratings with polarized light in azo dye doped POF [67,71,72,73,135,136]. In 2014, Hu et al. found that birefringent Bragg gratings were formed in POF gratings through the lateral inscription [47]. Further measurement in transmission with polarized light demonstrated that the photoinduced birefringence is ~7 × 10^−6^, similar to the one generated in photosensitive silica fibres. This feature could be advantageously used for transverse force sensing with POF gratings. In 2016, Oliveira et al. firstly reported the fast inscription of high-quality Bragg gratings in highly birefringent mPOFs by the phase mask method using 248 nm UV laser. The birefringent Bragg grating is deserved by a special design of the fibre structure [100].

#### 4.3.2. POF Gratings with Microstructure in Cladding

POF gratings with different microstructure in cladding have been fabricated by laser micromachining [129]. Firstly, PMMA SI POF contained a 5 mm long FBG, was fabricated by illuminating the fibre with 325 nm light through a phase mask placed on top of the POF. Then, the laser micromachined microstructures can be achieved by moving the POF grating under the static aperture laser beam and triggering the laser output, firing with a defined incremental distance movement of workpiece stage along the fibre. Multiple scan with laser radiation fluences of 1.37 J/cm^2^ and different masks have been used to generate the designed microstructures. Especially, POF gratings with slotted, open bay and D-shaped microstructure have been fabricated by the use of masks with a size of 400 μm, 800 μm and 1.6 mm, respectively.

#### 4.3.3. Etched/Micro POF Gratings

Besides POF gratings with microstructure in cladding, etched POF gratings were fabricated by the solvent etching after the grating inscription [148,149,150,151,152,153,154]. For further improvement of the response time and measurement sensitivity, micro POF gratings have also been fabricated based on two-stage process, where SM POF was etched to a certain diameter in the first instance, then tapered down by drawing to a micro POF [115], and finally inscribed Bragg gratings in it.

#### 4.3.4. Titled POF Gratings

In 2014, tilted fibre Bragg gratings (TFBGs) of TSB-doped photosensitive SI POF were inscribed by scanning phase mask at a velocity of 3 μm/s with a tilted phase mask in the plane perpendicular to the laser beam direction [45]. TFBG has 6 mm long and 3° titled. The reflected amplitude spectrum displays several narrow bands between ~1548 nm and ~1552 nm due to the multimode effect. Their FWHM does not exceed 100 pm, and the wavelength spacing between adjacent resonances lies in the range 0.4–0.7 nm.

#### 4.3.5. Multiplexed POF Grating Array

For multiplexed POF FBG array fabrication, the quickest way is adopting different phase masks and re-writing the grating in different region, but it will take more time for the phase mask swap and increase cost for the phase mask, so two alternative ways were proposed and used for the multiplex POF FBG array fabrication, which are the annealing technique and straining technique. It is known that when a POF is heated to a temperature where the polymer chains will start to relax from their orientation along the fibre, this causes the fibre to permanently shrink. Based on this thermal shrinkage, Johnson et al. inscribed two broadband FBGs in a large-core MM mPOF using a single phase mask, by thermally annealing the first grating before writing the next [88]. In the straining technique, it also uses a single phase mask but POF will be strained during the inscription, which is highly controlled tunning of the resonance wavelength of FBG. By straining the POF during writing, the resonance wavelength can linearly be tuned by 7 nm using only 1% strain [113]. Based on such a technique, dual-FBG strain sensor in mPOF and FBG array with five gratings have been fabricated [113,124] By comparison, the straining technique is much more controllable than the annealing technique and works with POFs regardless of their drawing conditions and whether they have been annealed or not. Especially, the tuning range with straining is about five times higher than that of silica fibres [155], which has great potential for future multiplexed sensing applications with POF gratings.

#### 4.3.6. POF Grating Based Fabry-Perot Cavity

Dobb et al. fabricated the first Fabry-Perot cavity in PMMA SI POF, which is formed from two 1 cm long FBGs separated by 3 cm. The approximately sinusoidal nature of the fringes superimposed on the grating reflection spectrum is indicative of a low finesse cavity, caused partially by the large attenuation at ~1570 nm [107,108].

#### 4.3.7. Phase Shifted POF Gratings

A π-shifted grating in PMMA SI POF was produced by the uniform exposure to UV of the central 1 mm section of a 1 cm long FBG [107,108]. However, it lacked sharpness in the central reflection notch partially due to the high attenuation. A move to gratings designed for visible wavelengths should result in a much sharper spectral feature and the correspondingly higher precision [107,108].

#### 4.3.8. Reversible POF Gratings 

Based on the reversible photosensitive mechanism of azobenzene [67,69,71,72,73,74] and Photosol 7-049 [56], reversible POF gratings could be written-erased-written in several cycles. Such reversible gratings have great potential applications in all-optical controlled optical add-drop multiplexer and other intelligent, photo-addressable and reconfigurable system.

#### 4.3.9. Chirped POF Gratings

Although linearly chirped POF Bragg gratings made in fibre tapers have been proposed for the dispersion tuning without center wavelength shift as early as 2005 [156], there is no report for the fabrication of the chirped POF gratings.

## 5. Final Remarks

Significant progress has been made in research, development and application of POF gratings since the first demonstration of POF gratings in 1999. POF gratings have successfully demonstrated, with various fabrication techniques including different sources and wavelengths ranged from 248 to 800 nm, and in various host materials, ranging from PMMA to COC with low moisture absorption, to CYTOP with low loss, and to PC with high glass transition temperature. Progress has been made in better understanding of key issues such as material photosensitivity and writing wavelength and power in fabricating POF gratings. The wavelength of 325 nm remains the most popular writing wavelength (now often provided by a commercial He-Cd laser) for the POF gratings fabrication, although many different writing wavelengths have tried and reported. For easier fabrication of POF gratings, a variety of photosensitive POFs have been developed through the introduction of different dopants by either simple doping or co-polymerization. In addition, different types of POF gratings have been fabricated, including Bragg gratings, LPGs as well as many types of special POF gratings and the operating wavelength of POF gratings has extended from NIR band (~1550 nm) with high loss to visible band (~633 nm) nearly located at the low-loss transmission window of POF. As evidence of significant progress for practical applications, the inscription time has reduced from previously in tens of minutes to currently in seconds and the bandwidth of POF gratings has decreased from 1 nm to 0.045 nm. These are important progress for easier, better and more diverse production of POF gratings for future photonic applications.

## Figures and Tables

**Table 1:** The properties of host materials for POF gratings.

Materials	Structure	n	T_g_	T_m_	α	dn/dT	Stress-Optic Coefficient	Moisture Absorption
			°C	°C	10^−4^ °C^−1^	10^−4^ °C^−1^	10^−12^ Pa^−1^	wt %
poly(methyl methacrylate) (PMMA)	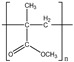	1.49 [31]	104 [31]	160 [31]	0.68 [31]	−1.05 [31] −1.2 [31]	−4.5~−1.5 [31]	up to 2.0 [31]
polycarbonates (PC)	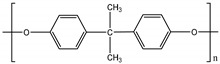	1.58 [31]	170 [31]	267 [31]	0.66 [31]	−1.07 [31] −1.1~−1.4 [31]	68 [31]	0.04 [31]
polystyrene (PS)	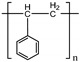	1.59 [31]	~90 [31]	240 [31]	0.7 [32]	−1.3 [33]	4.8 [31]	0.1–0.3 [31]
cyclic olefin copolymer (COC, TOPAS)	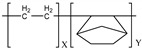	1.53 [31]	70–177 [31]	190–320 [31]	0.6 [31]	−1.0 [31]	4.0 [31]	0.01 [31]
amorphous fluoropolymer (CYTOP, Asahi Glass)	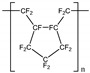	1.34 [31]	108 [31]	-	1.15–1.20 [31]	−0.97 [34]	6.5 [35]	<0.01 [35]
poly(ethyl methacrylate) (PEMA)	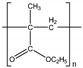	1.485 [36]	63 [36]	-	0.6 [37]	−1.1 [38]	-	0.5-2.0 [37]
poly(benzyl methacrylate) (PBzMA)	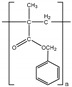	1.568 [39]	54 [39]	-	1.7 [40]	-	4.0 [40]	-
poly(butyl acrylate) (PBA)	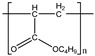	1.47 [41]	−53 [41]	45 [42]	-	-	−0.45 [42]	-
poly(trifluroethyl ethacrylate) (PFMA)	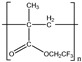	1.418 [43]	69 [43]	51 [43]	-	-	-	-

**Table 2:** Photosensitive POFs developed by several research groups.

Research Group	Photosensitive POF	Reference
University of New South Wales, Australia	Fluorescein, Rhodamine 6G-doped POF	[22,77]
University of Science & Technology of China (USTC), China	Azobenzene-, BDK-, VA-, MVK-doped POF	[65,66,67,75]
The Hong Kong Polytechnic University, Hong Kong, China	TSB-doped POF and mPOF	[64,97]
Aston University, UK	BDK-doped mPOF	[68]

**Table 3:** Sources and mechanisms of fabricating POF gratings.

Source	Wavelength	Mechanism	Reference
UV mercury lamp	multi lines	Photo-degradation	[75]
KrF excimer laser	248 nm	-	[49,99,100,101,102]
XeCl excimer laser	308 nm	-	[46]
UV laser OPO pulsed laser Dye laser He-Cd laser	325 nm	photocrosslinking photopolymerization photoisomerization photolock polymer chain degradation photoblation due to high absorption	[22,44,45,47,52,53,54,57,60,64,68,70,77,82,85,86,87,88,89,90,91,92,95,103,104,105,106,107,108,109,110,111,112,113,114,115,116,117,118,119,120,121,122,123,124,125,126,127,128,129]
Nd:YAG laser	355 nm	polymerization photolock polymer chain degradation photocrosslinking	[48,65,66,76,130]
Ti: sapphire fs laser	387 nm	ultrashort photo-modification-polymer backbone cleavage & monomer production photocrosslinking	[78,79,80,131]
Ti: sapphire fs laser	400 nm	refractive index modification via 2-photon absorption	[84]
He-Cd laser	421.8 nm	photoinduced birefringence	[69,74]
Ar^+^ laser	501.7 nm	optical ring cleavage	[56]
Ar^+^ laser	514 nm	-	[132]
fs laser	517 nm	-	[133,134]
Nd:YVO_4_ laser	532 nm	photoinduced birefringence	[67,71,72,73,135,136]
Ti:sapphire fs laser	800 nm	refractive index modifications via 2-photon absorption	[81,137,138,139,140]

**Table 4:** The fabrication conditions and the main characteristics of LPGs in POF.

Core	Cladding	Dopant	POF	Source	Writing Method	Λ	L	Loss Dip	FWHM	λ_r_	Reference
			type			μm	mm	dB	nm	nm	
PMMA	-	azobenzene dye (800 ppm)	-	532 nm laser	Talbot effect	400	-	-	-	-	[135,136]
PMMA	-	-	SM mPOF	-	Stable imprint	1000	150	34	3	570	[147]
P(MMA-co-MVK-co-BzMA)	PMMA	MVK	SI	UV mercury lamp	Amplitude mask	275	30	3	3	1568	[75]
P(MMA-co-BA-CAMA)	P(MMA-co-BA)	CAMA (3–4 wt %)	SI	532 nm Nd:YVO_4_ laser	Amplitude mask	120	-	-	-	-	[67,71,72,73]
PMMA	-	-	SM mPOF	-	Stable imprint	500 2620	150	16	20 10	510	[142]
PMMA	-	-	SM SI	387 nm Ti: sapphire fs laser	Interference	50	-	-	-	-	[78,79]
						1.4					
PMMA	-	-	MM SI	400 nm fs laser	Point-by-point	189	-	-	-	-	[84]
PMMA	-	-	SM SI	800 nm fs laser	Point-by-point	~1.0	-	-	-	-	[138]
PMMA	-	-	mPOF	325 nm He-Cd laser	Point-by-point	1000	20	9	15	840	[109,110]
Poly(MMA-co-EMA-co-BzMA)	Poly(MMA–BMA)	TSB (0.66 wt %)	MM SI	325 nm dye laser	Phase mask	-	-	-	-	-	[87]
Poly(MMA-co-BA-co-9-VA)	Poly(MMA-co-BA)	VA (0.2 mol %)	MM SI	pulsed 355 nm laser	Point-by-point	836	42.6	12.8	10	1530	[76]
CYTOP	PMMA	-	MM GI	355 nm Nd:YAG	Point-by-point	75	7.5	25	~0.8	910	[48]
PMMA	-	TSB	mPOF	325 nm He-Cd laser	Point-by-point	1000	10	20	45	830	[123]
PMMA	-	Azobenzene	mPOF	325 nm He-Cd laser	Point-by-point	1200	19.2	15	22	778	[82]

Note: Λ, L and λ_r_ are the period of gratings, length of the grating region and resonance wavelength.

## Supplementary Materials

The following are available online at http://www.mdpi.com/1424-8220/17/3/511/s1, Table S1: POF materials and their photosensitivity.
