# Supplementary material for: Fabrication of Polymer Optical Fibre (POF) Gratings
Source: Sensors (Basel). 2017 Mar 4;17(3):511. doi: 10.3390/s17030511 (PMC5375797; doi:10.3390/s17030511)
Supplement: Supplementary file 1 [file sensors-17-00511-s001.pdf]

sensors

Supporting Information

Fabrication of Polymer Optical Fibre (POF) Gratings

Yanhua Luo[1,2], Binbin Yan[3], Qijin Zhang[4], Gang-Ding Peng[1,*] Jianxiang Wen[5] and Jianzhong Zhang[6]

[1] Photonics & Optical Communications, School of Electrical Engineering and Telecommunications, University of New South Wales, Sydney, NSW 2052, Australia; yanhua.luo1@unsw.edu.au; g.peng@unsw.edu.au
[2] State Key Laboratory for Modification of Chemical Fibers and Polymer Materials, Donghua University, Shanghai 201600, China;
[3] State Key Laboratory of Information Photonics and Optical Communications, Beijing University of Posts and Telecommunications, Beijing 100876, China; yanbinbin@bupt.edu.cn
[4] CAS Key Laboratory of Soft Matter Chemistry, Department of Polymer Science and Engineering, University of Science and Technology of China, Hefei, Anhui 230026, China; zqjm@ustc.edu.cn
[5] Key Laboratory of Specialty Fiber Optics and Optical Access Networks, Shanghai University, Shanghai 200072, China; wenjx@shu.edu.cn
[6] Key Lab of In-fiber Integrated Optics, Ministry of Education, Harbin Engineering University, Harbin 150001, China; zhangjianzhong@hrbeu.edu.cn

* Correspondence: g.peng@unsw.edu.au; Tel.: +61-2-9385 4014

Table S1. POF materials and their photosensitivity

Core	Cladding	Dopant	POF	Source	Power	Time	Δn	Mechanism	Ref
PMMA	-	-	-	325 nm UV laser	6 mW (focused)	-	3×10^{-3}	photoinduced cross linking	[1]
Poly(BzMA-co-MMA)	P(EMA-co-MMA)	fluorescein (170ppm)	two core SI	514 nm Ar^{+} laser	0.36-1.08 mW	62 mins	3.3×10^{-5}	-	[2]
Poly(BzMA-co-MMA)	P(EMA-co-MMA)	fluorescein (170ppm)	two core SI	OPO pulsed laser @ 248, 280, 325 nm	-	-	-	-	[2]
Poly(BzMA-co-MMA)	P(EMA-co-MMA)	Rhodamine 6G	MM SI	325 nm OPO pulsed laser	-	-	-	-	[3]
Poly(MMA-co-EMA-co-BzMA)	PMMA	-	SM SI	325 nm OPO pulsed laser	-	-	10^{-4}	photo cross-linking & photopolymerization	[4]
CYTOP	-	-	-	355 nm Nd:YAG laser	350 mJ	20 mins	3×10^{-4}	-	[5]
PMMA	-	azobenzene dye(800ppm)	MM SI	532nm Nd:YVO_4 laser	87mW/cm^2	60mins	3×10^{-5}	photo induced birefringence	[6-7]
Poly(MMA-co-EMA-co-BzMA)	PMMA	-	SM SI	325nm OPO pulsed laser	-	85 mins	1×10^{-3}	-	[8-10]
PMMA	-	-	-	800nm 40 fs Ti:sapphire	1 J/cm^2	-	5×10^{-4}	-	[11]
Poly(MMA-co-CAMA)	Poly(MMA-co-BA)	CAMA (3-4 wt%)	SI	532 nm Nd:YVO_4 laser	-	-	-	photo-induced birefringence	[12-15]
Poly(MMA-co-EMA-co-BzMA)	Poly(MMA-co-BMA)	TSB (0.66wt%)	MM SI	325 nm laser	0.208 W/ cm^2	10 mins	-3×10^{-4}	photoisomerization	[16]
PMMA (doped DPS 0.5%)	PMMA	Photosol 7-049 (0.5%)	MM GI	501.7 nm Ar laser	4 mW	-	-	optical ring cleavage	[17]
Poly(MMA-co-EMA-co-BzMA)	PMMA	-	SM SI	325 nm OPO laser	4.5 mJ	450 mins	2.6×10^{-4}	-	[18]
Poly(MMA-co-MVK-co-BzMA)	PMMA	MVK (8wt%)	FM SI	UV mercury lamp	0.3 mW/cm^2	200 s	6×10^{-3} (10 wt%)	photodegradation	[19]

**Core**	**Cladding**	**Dopant**	**POF**	**Source**	**Power**	**Time**	**Δn**	**Mechanism**	**Ref**
PMMA	-	-	FM & SM mPOF	325 nm He-Cd laser		60 mins	-	-	[20]
Poly(BzMA-co-MMA)	P(EMA-co-MMA)	-	SI	325 nm He-Cd laser	30 mW	-	-	-	[21-22]
PMMA	-	-	SM SI	387 nm Ti: sapphire fs laser	0.8J/cm^2	-	2×10^{-3}	polymer backbone cleavage & monomer production	[23-25]
Poly(MMA-co-CAMA)	Poly(MMA-co-BA)	CAMA (3.2 wt%)	SI	421.8 nm He-Cd laser	-	-	-	photoinduced birefringence	[26-27]
PMMA	-	-	MM & SM SI	400 nm fs laser	80-100nJ	-	-	index change via 2-photon absorption	[28]
PMMA-co-PS (5% PS)	PMMA	-	SM SI	308 nm XeCl excimer laser	85 mJ/cm^2	60 mins	-	-	[29]
Poly(MMA-co-EMA-co-BzMA)	Poly(MMA–EMA)	-	SM SI	308 nm XeCl excimer laser	70 mJ/cm^2	17 mins	-	-	[29]
PMMA	-	-	POF	800 nm fs laser	60 nJ	-	-	refractive index modifications	[30]
PMMA	-	-	mPOF	325 nm He-Cd laser	30 mW	-	-		[31-32]
PMMA	-	-	-	387 nm fs laser	0.446 J/cm^2	-	5×10^{-5}	depolymerization and crosslinking	[33]
Poly(MMA-co-EMA-co-BzMA)	Poly(MMA-co-EMA)	BDK (2wt%)	MM &SM SI	355 nm Nd:YAG	0.673 W/cm^2	16 mins	4.5×10^{-5}	polymerization photolock & photodegradation	[34]
Poly(MMA-co-EMA-co-BzMA)	Poly(MMA-co-BMA)	TSB (1wt%)	MM SI	325 nm laser	-	15 mins	1.5×10^{-5}	photoisomerization	[35]
PMMA	-	-	MM mPOF	325 nm He-Cd laser	30 mW	-	-	-	[36-38]
Poly(MMA-co-EMA-co-BzMA)	Poly(MMA-co-BMA)	TSB (1wt%)	MM SI	325 nm laser	-	-	-	photoisomerization	[39]

**Core**	**Cladding**	**Dopant**	**POF**	**Source**	**Power**	**Time**	**Δn**	**Mechanism**	**Ref**
COC	-	-	FM mPOF	325 nm He-Cd laser	30 mW	30-40 mins	-	-	[40]
COC	-	-	SM mPOF	325 nm He-Cd laser	30 mW	-	-	-	[41]
COC	-	-	mPOF	325 nm He-Cd laser	5 W/cm^2	300 mins	-	-	[42]
PMMA	-	-	FM mPOF	325 nm He-Cd laser	-	185 mins	-	-	[43]
PMMA-co-PS (5% PS)	PMMA	-	FM SI	325 nm He-Cd laser		60 mins	-	-	[43]
COC	-	-	mPOF	325 nm He-Cd laser	5 W/cm^2	338 mins	1.5×10^{-5}		[44]
Poly(MMA-co-BA-co-VA)	Poly(MMA-co-BA)	VA (0.2 mol%)	FM SI	355 nm laser	10 mW	-	8.0×10^{-4}	photo-crosslinking	[45-46]
PMMA	-	BDK	mPOF	325 nm He-Cd laser	2.65 W/cm^2	13 mins	3.2×10^{-4}	polymerization, photolock, & photodegradation	[47]
COC	-	-	-	248 nm KrF excimer laser	-	30 s	-	-	[48]
COC	-	-	-	248 nm KrF excimer laser	-	8 s	-	-	[48]
PMMA	-	-	mPOF	800 nm fs laser	-	2.5 s	-		[49-50]
PMMA	-	-	mPOF	325 nm He-Cd laser	-	-	-		[51]
PMMA	-	-	-	325 nm He-Cd laser	-	-	-	-	[52]
CYTOP	PMMA	-	MM GI	355 nm Nd:YAG laser	-	-	-	-	[53]
PMMA-co-PS (5% PS)	PMMA	-	SM SI	248 nm laser	0.5 mJ/cm^2	-	-		[54]
Poly(MMA-co-EMA-co-BzMA)	Poly(MMA-co-EMA)	-	micro POF	325 nm He-Cd laser	50 mW	3 mins	-		[55]

**Core**	**Cladding**	**Dopant**	**POF**	**Source**	**Power**	**Time**	**Δn**	**Mechanism**	**Ref**
PMMA	-	-	SM POF	325 nm He-Cd laser	30 mW	-	-		[56]
PMMA	-	-	SM mPOF	325 nm He-Cd laser	30 mW	-	-		[57-59]
PMMA-co-PS (5% PS)	PMMA	-	SM SI POF	325 nm He-Cd laser	30 mW	-	-		[57-59]
PMMA	-	-	SM mPOF	325 nm He-Cd laser	30 mW	-	-		[57-59]
PMMA-co-PS (5% PS)	PMMA	-	SM SI POF	325 nm He-Cd laser	30 mW	-	-		[57-59]
PMMA	-	-	SM & MM mPOF	325 nm He-Cd laser	30 mW	-	-		[57-59]
PMMA	-	-	mPOF	325 nm He-Cd laser	30 mW	7 mins	-	-	[60-61]
CYTOP	?	-	MM GI	248 nm KrF excimer laser	5k J/cm^2	-	-	-	[62]
PMMA	-	-	mPOF	325 nm He-Cd laser	900 kW/m^2	-	8.5×10^{-3}	Photodegradation & further polymerization	[63-64]
PMMA	-	TSB	mPOF	325 nm He-Cd laser	30 mW	42 s	-	photoblation due to high absorption	[65]
PMMA(doped DPS 5 mol%)	PMMA	TSB (1wt%)	SI POF	325 nm He-Cd laser	-	-	-	photoisomerization	[66]
PMMA(doped DPS 5 mol%)	PMMA	TSB (1wt%)	SI POF	325 nm He-Cd laser	-	-	-	photoisomerization	[67]
Poly(MMA-co-EMA-co-BzMA)	Poly(MMA-co-EMA)	-	SM SI	325 nm He-Cd laser	50 mW	-	-		[68]
CYTOP	PMMA	-	MM GI	325 nm He-Cd laser	30 mW	12 mins	-		[69]
PMMA	-	-	SM mPOF	325 nm He-Cd laser	30 mW	50 mins	-		[69]

**Core**	**Cladding**	**Dopant**	**POF**	**Source**	**Power**	**Time**	**Δn**	**Mechanism**	**Ref**
PC	-	-	SM mPOF	325 nm He-Cd laser	50 mW	-	-	-	[70]
CYTOP	PE/PC	-	MM GI	517 nm fs laser	~80 nJ/pulse	-	1.3×10^{-4}	-	[71-72]
COC (TOPAS 5013S-04)	COC (ZEONEX 480R)	-	SM SI	325 nm He-Cd laser	-	-	-	-	[73]
PC	-	-	SM mPOF	325 nm He-Cd laser	4 mW	~6 mins	-	-	[74]
PMMA	-	-	SM mPOF	325 nm He-Cd laser	30 mW	15 mins		-	[75-76]
PMMA	-	-	SM mPOF	325 nm He-Cd laser	20 mW	-	-		[77]
PMMA	-	-	FM mPOF	248 nm laser	33 mJ/cm^2	30 s	2.4×10^{-4}		[78]
PMMA	-	-	D-shape SM SI	325 nm He-Cd laser	-	-	-		[79]
PMMA	-	azobenzene	mPOF	325 nm He-Cd laser	30 mW	42 s	-	photoblation due to high absorption	[80]
PMMA (doped DPS 5mol%)	PMMA	TSB (1wt%)	SI	800 nm fs laser	20 mW	70 s	-	index change via 2-photon absorption	[81]
COC (TOPAS 5013S-04)	COC (ZEONEX 480R)	-	SM SI	325 nm He-Cd laser	6 mW	4 mins			[82]
PMMA	-	-	HiBi mPOF	248 nm KrF laser	33 mJ/cm^2		-		[83]
Poly(MMA-co-EMA-co-BzMA)	Poly(MMA-co-EMA)	-	micro POF	325 nm He-Cd laser	50 mW	7 s			[84]

Note: CAMA: 2-{4-[2-(4-cyano-phenyl)-diazenyl]–phenyloxy}-ethyl methacrylate

References

1. Tomlinson, W. J.; Kaminow, I. P.; Chandross, E. A.; Fork, R. L.; Silfvast, W. T. Photoinduced refractive index increase in poly(methylmethacrylate) and its applications. *Appl. Phys. Lett.* **1970**, *16*, 486-489.
2. Chu, P. L.; Peng, G. D. Photosensitivities in germanium-doped planar waveguides and dye-doped polymer optical fibres. *Proc. SPIE* **1998**, *3470*, 120-127.
3. Peng, G. D.; Xiong, Z.; Chu, P. L. Photosensitivity and gratings in dye-doped polymer optical fibers. Opt. Fiber Technol. **1999**, 5, 242-251.
4. Xiong, Z.; Peng, G. D.; Wu, B.; Chu, P. L. Highly tunable Bragg gratings in single-mode polymer optical fibers. *IEEE Photon. Technol. Lett.* **1999**, *11*, 352-354.
5. Liu, H. Y.; Peng, G. D.; Chu, P. L.; Koike, Y.; Watanabe, Y. Photosensitivity in low-loss perfluoropolymer (CYTOP) fibre material. *Electron. Lett.* **2001**, *37*, 347-348.
6. Xu, X.; Ming, H.; Ma, H.; Sun, X.; Cheng, W.; Ye, J.; Zhang, Q.; Xie, J. Birefringent gratings induced by polarized laser in azobenzene-doped poly(methyl methecrylate) optical fibers. *Proc. SPIE* **2001**, 4603, 260-265.
7. Xu, X.; Ming, H.; Zhang, Q. Properties of polarized laser-induced birefringent gratings in azobenzene-doped poly(methyl methecrylate) optical fibers. *Opt. Commun.* **2002**, *204*, 137-143.
8. Liu, H. Y.; Peng, G. D.; Chu, P. L. Polymer fiber Bragg gratings with 28-dB transmission rejection. *IEEE Photon. Technol. Lett.* **2002**, *14*, 935-937.
9. Peng, G.-D.; Liu, H.; Chu, P. L. Dynamics and threshold behaviour in polymer fibre bragg grating creation. *Proc. SPIE* **2002**, *4803*, 164-178.
10. Peng, G.; Liu, H. Y.; Chu, P. L. Highly reflective polymer fiber bragg gratings and its growth dynamics. In *Optical Fiber Communications Conference*, Anaheim, California, 17 Mar. 2002; OSA.
11. Scully, P. J.; Jones, D.; Jaroszynski, D. A. Femtosecond laser irradiation of polymethylmethacrylate for refractive index gratings. *J. Opt. A-Pure Appl. Opt.* **2003**, *5*, S92–S96.
12. Ma, H.; Li, Z.-C.; Ming, H.; Zhang, Q.; Tam, H.-Y.; Zhang, Y.-S.; Zhang, T.; Wang, P.; Xie, J. Analysis of photosensitivity of copolymer optical fibre preform. *Chin. Phys. Lett.* **2004**, *21*, 2252-2254.
13. Li, Z.; Ma, H.; Zhang, Q.; Ming, H. Birefringence grating within a single mode polymer optical fibre with photosensitive core of azobenzene copolymer. *J. Optoelectron. Adv. Mater.* **2005**, *7*, 1039-1046.
14. Zheng, R. S.; Lu, Y. H.; Xie, Z. G.; Tao, J.; Lin, K. Q.; Ming, H. Surface plasmon resonance sensors based on polymer optical fiber. In 1st Asia-Pacific Optical Fiber Sensors Conference, Chengdu, China, 7-9 Nov. 2008; IEEE.
15. Luo, Y.; Li, Z.; Zheng, R.; Chen, R.; Yan, Q.; Zhang, Q.; Peng, G.; Ming, H.; Zhu, B. Birefringent azopolymer long period fiber gratings induced by 532 nm polarized laser. *Opt. Commun.* **2009**, *282*, 2384-2353.
16. Yu, J.; Tao, X.; Tam, H. Trans-4-stilbenemethanol-doped photosensitive polymer fibers and gratings. *Opt. Lett.* **2004**, *29*, 156-158.
17. Boxel, R. V.; Verbiest, T.; Persoons, A. Switchable Bragg gratings in photochromic doped graded-index polymer optical fibres. *Proc. SPIE* **2004**, *5279*, 77-84.
18. Liu, H. B.; Liu, H. Y.; Peng, G. D.; Chu, P. L. Novel growth behaviors of fiber Bragg gratings in polymer optical fiber under UV irradiation with low power. *IEEE Photon. Technol. Lett.* **2004**, *16*, 159-161.
19. Li, Z.; Tam, H. Y.; Xu, L.; Zhang, Q. Fabrication of long-period gratings in poly(methyl methacrylate-co-methyl vinyl ketone-co-benzyl methacrylate)-core polymer optical fiber by use of a mercury lamp. *Opt. Lett.* **2005**, *30*, 1117-1119.
20. Dobb, H.; Webb, D. J.; Kalli, K.; Argyros, A.; Large, M. C. J.; van Eijkelenborg, M. A. Continuous wave ultraviolet light-induced fiber Bragg gratings in few- and single-mode microstructured polymer optical fibers. *Opt. Lett.* **2005**, *30*, 3296-3298.
21. Webb, D. J.; Aressy, M.; Argyros, A.; Barton, J. S.; Dobb, H.; Eijkelenborg, M. A. v.; Fender, A.; Jones, J. D. C.; Kalli, K.; Kukureka, S.; Large, M. C. J.; MacPherson, W.; Peng, G. D.; Silva-López, M. Grating and interferometric devices in POF. In *14th International Polymer Optical Fibre Conference*, Hong Kong, China, 19-21 September 2005; Optoelectronics Research Centre City Univeristy of Hong Kong, pp.325-328.
22. Dobb, H.; Carroll, K.; Webb, D. J.; Kalli, K.; Komodromos, M.; Themistos, C.; Peng, G. D.; Argyros, A.; Large, M. C. J.; Eijkelenborg, M. A. v.; Fang, Q.; Boyd, I. W. Grating based devices in polymer optical fibre. *Proc. SPIE* **2006**, *6189,* 618901-1-12.
23. Baum, A.; Scully, P. J.; Perrie, W. Femtosecond Laser Modification of Poly(methyl methacrylate) at 387 nm Wavelength In *Conference on Lasers and Electro-Optics*, Long Beach, California, 21 May 2006; OSA.
24. Baum, A.; Perrie, W.; Scully, P. J.; Basanta, M.; Thomas, C. L.; Goddard, N. J.; Fielden, P. R.; Chalker, P. Refractive index structures in poly(methyl methacrylate) and polymer optical fibre by femtosecond laser

irradiation in *optical fiber sensors*, Cancun, 23 Oct. 2006; OSA.

25. Baum, A.; Scully, P. J.; Basanta, M.; Paul Thomas, C. L.; Fielden, P. R.; Goddard, N. J.; Perrie, W.; Chalker, P. R. Photochemistry of refractive index structures in poly(methyl methacrylate) by femtosecond laser irradiation. *Opt. Lett.* 2007, *32*, 190-192.
26. Luo, Y.; Zhou, J.; Yan, Q.; Su, W.; Li, Z.; Zhang, Q.; Huang, J.; Wang, K. Optical manipulable polymer optical fiber Bragg gratings with azopolymer as core material. *Appl. Phys. Lett.* 2007, *91*, 071110.
27. Luo, Y.; Wu, W.; Yan, Q.; Zhang, Q.; Peng, G.-D. Reversible birefringent gratings in azo polymer optical fibre. In *The 18th International Conference on Plastic Optical Fibers*: Sydney, Australia, 9-11 Sep. 2009.
28. Liang, S. J.; Scully, P. J.; Schille, J.; Vaughan, J.; Perrie, W. Femtosecond laser induced refractive index structures in polymer optical fibre (POF) for sensing. *Proc. SPIE* 2009, *7503*, 75036S-1-4.
29. Terblanche, J.; Schmieder, D.; Meyer, J. Fibre Bragg gratings in polymer optical fibres at 980 nm. *Proc. SPIE* 2009, *7503*, 75037F-1-4.
30. Stecher, M.; Williams, R. J.; Bang, O.; Marshall, G. D.; Withford, M. J.; Town, G. E. Periodic refractive index modifications inscribed in polymer optical fibre by focussed femtosecond pulses, In *The 18th International Conference on Plastic Optical Fibers*: Sydney, Australia, 9-11 Sep. 2009.
31. Sáez-Rodríguez, D.; Munoz, J. L. C.; Johnson, I.; Webb, D. J.; Large, M. C. J.; Argyros, A. Long period fibre gratings photoinscribed in a microstructured polymer optical fibre by UV radiation. *Proc. SPIE* 2009, *7357*, 73570L-1-8.
32. Saez-Rodriguez, D.; Cruz, J. L.; Johnson, I.; Webb, D. J.; Large, M. C. J.; Argyros, A. Water diffusion into UV inscripted long period grating in microstructured polymer fiber. *IEEE Sens. J.* 2010, *10*, 1169-1173.
33. Baum, A.; Scully, P. J.; Perrie, W.; Liu, D.; Lucarini, V. Mechanisms of femtosecond laser-induced refractive index modification of poly(methyl methacrylate). *J. Opt. Soc. Am. B-Opt. Phys.* **2010**, *27*, 107-111.
34. Luo, Y.; Zhang, Q.; Liu, H.; Peng, G. Grating fabrication in benzildimethylketal doped photosensitive polymer fibers using 355 nm nanosecond pulsed laser. *Opt. Lett.* **2010**, *35*, 751-753.
35. Zhang, Z. F.; Zhang, C.; Tao, X. M.; Wang, G. F.; Peng, G. D. Inscription of polymer optical fiber bragg grating at 962 nm and its potential in strain sensing. *IEEE Photon. Technol. Lett.* **2010**, *22*, 1562-1564.
36. Johnson, I. P.; Webb, D. J.; Kalli, K.; Large, M. C. J.; Argyros, A. Multiplexed FBG sensor recorded in multimode microstructured polymer optical fibre. *Proc. SPIE* **2010**, *7714*, 77140D-1-10.
37. Johnson, I. P.; Webb, D. J.; Kalli, K. Utilisation of thermal annealing to record multiplexed FBG sensors in multimode microstructured polymer optical fibre. *Proc. SPIE* **2010**, *7753*, 77536T-1-4.
38. Johnson, I. P.; Webb, D. J.; Kalli, K.; Yuan, W.; Stefani, A.; Nielsen, K.; Rasmussen, H. K.; Bang, O. Polymer PCF Bragg grating sensors based on poly(methyl methacrylate) and TOPAS cyclic olefin copolymer. *Proc. SPIE* **2011**, *8073*, 80732V-1-8.
39. Sun, X.; Tao, X.; Zhang, Z. The investigation on photosensitivity of polymer optical fiber. In *2011 Symposium on Photonics and Optoelectronics*, Wuhan, China, 16-18 May 2011; IEEE.
40. Zhang, C.; Webb, D.; Kalli, K.; Emiliyanov, G.; Bang, O.; Kjær, E. Bragg grating inscription in TOPAS microstructured polymer optical fibre. In 16th International Conference on Plastic Optical Fibers, Turin, Italy, 10-12 Sep. 2007.
41. Johnson, I. P.; Yuan, W.; Stefani, A.; Nielsen, K.; Rasmussen, H. K.; Khan, L.; Webb, D. J.; Kalli, K.; Bang, O. Optical fibre Bragg grating recorded in TOPAS cyclic olefin copolymer. *Electron. Lett.* **2011**, *47*, 271-272.
42. Yuan, W.; Khan, L.; Webb, D. J.; Kalli, K.; Rasmussen, H. K.; Stefani, A.; Bang, O. Humidity insensitive TOPAS polymer fiber Bragg grating sensor. *Opt. Express* **2011**, *19*, 19731-19739.
43. Stefani, A.; Yuan, W.; Markos, C.; Bang, O. Narrow bandwidth 850 nm fiber Bragg gratings in few-mode polymer optical fibers. *IEEE Photon. Technol. Lett.* **2011**, *23*, 660-662.
44. Yuan, W.; Webb, D. J.; Kalli, K.; Nielsen, K.; Stefani, A.; Rasmussen, H. K.; Bang, O. 870nm Bragg grating in single mode TOPAS microstructured polymer optical fibre. *Proc. SPIE* **2011**, *7753*, 77538X-1-4.
45. Wang, T.; Wang, Q.; Luo, Y.; Qiu, W.; Peng, G.-D.; Zhu, B.; Hu, Z.; Zou, G.; Qijin Zhang. Enhancing photosensitivity in near UV/vis band by doping 9-vinylanthracene in polymer optical fiber. *Opt. Commun.* **2013**, *307*, 5-8.
46. Wang, Q.; Wang, T.; Qiu, W.; Luo, Y.; Zhang, Q.; Zhu, B. Long period fiber gratings in 9-vinylanthracene-containing core of photosensitive polymer optical fiber. In *Asia Communications and Photonics Conference*, Guangzhou, China, 07 Nov. 2012; OSA.
47. Sáez-Rodríguez, D.; Nielsen, K.; Rasmussen, H. K.; Bang, O.; Webb, D. J. Highly photosensitive polymethyl methacrylate microstructured polymer optical fiber with doped core. *Opt. Lett.* **2013**, *38*, 3769-3772.
48. Zhou, S. F.; Reekie, L.; Chan, H. P.; Chow, Y. T.; Chung, P. S.; Man Luk, K. Characterization and modeling of Bragg gratings written in polymer fiber for use as filters in the THz region. *Opt. Express* **2012**, *20*, 9564-9571.

49. Stefani, A.; Stecher, M.; Town, G. E.; Bang, O. Fiber design and realization of point-by-point written fiber Bragg gratings in polymer optical fibers. *Proc. SPIE* **2012**, *8426*, 842617-1-7.
50. Stefani, A.; Stecher, M.; Town, G. E.; Bang, O. Direct writing of fiber Bragg grating in microstructured polymer optical fiber. *IEEE Photon. Technol. Lett.* **2012**, *24*, 1148-1150.
51. Yuan, W.; Stefani, A.; Bang, O. Tunable polymer fiber Bragg grating (FBG) inscription: fabrication of dual-fbg temperature compensated polymer optical fiber strain sensors. *IEEE Photon. Technol. Lett.* **2012**, *24*, 401-403.
52. Khan, L.; Webb, D.; Kalli, K. Potential for broad range multiplexing of fibre Bragg gratings in polymer optical fibres and operation in the 700-nm wavelength range, In *Micro-structured and specialty optical fibres II*, Prague, Czech Republic, 15-18 Apr. 2013; SPIE.
53. Castrellon-Uribe, J.; Lomer, M.; Roufael, H.; Lopez-Higuera, J. M. Efficient PF-POF external LPG index Transducer. In *Workshop on Specialty Optical Fibers and their Applications*, Sigtuna, 28 Mar. 2013; OSA.
54. Ramakrishnan, R.; Mathews, S.; Rajan, G.; Semenova, Y.; Gerald, F.; Srinivasan, B. Fabrication and characterization of Bragg gratings in polymer optical fibers using 248 nm irradiation. In *Asia Communications and Photonics Conference 2013*, Beijing, China, 12 Nov. 2013; OSA.
55. Rajan, G.; Noor, M. Y. M.; Lovell, N. H.; Ambikaizrajah, E.; Farrell, G.; Peng, G.-D. Polymer micro-fiber Bragg grating. *Opt. Lett.* **2013**, *38*, 3359-3362.
56. Statkiewicz-Barabach, G.; Tarnowski, K.; Kowal, D.; Mergo, P.; Urbanczyk, W. Fabrication of multiple Bragg gratings in microstructured polymer fibers using a phase mask with several diffraction orders. *Opt. Express* **2013**, *21*, 8521-8534.
57. Carlos, A. F. M.; Lúcia, B. B.; Nélia, J. A.; David, J. W.; Rogério, N. N. Inscription of narrow bandwidth Bragg gratings in polymer optical fibers. *J. Opt.* **2013**, *15*, 075404.
58. Marques, C. A. F.; Bilro, L. B.; Alberto, N. J.; Webb, D. J.; Nogueira, R. N. Narrow bandwidth Bragg gratings imprinted in polymer optical fibers for different spectral windows. *Opt. Commun.* **2013**, *307*, 57-61.
59. Marques, C. A. F.; Bilro, L.; Webb, D. J.; Nogueira, R. N. Inscription of narrow bandwidth Bragg gratings in polymer optical fibers. *Proc. SPIE* **2013**, *8794*, 879423-1-4.
60. Bundalo, I.-L.; Nielsen, K.; Markos, C.; Bang, O. Bragg grating writing in PMMA microstructured polymer optical fibers in less than 7 minutes. *Opt. Express* **2014**, *22*, 5270-5276.
61. Bundalo, I.-L.; Nielsen, K.; Markos, C.; Bang, O. PMMA mPOF Bragg gratings written in less than 10 min. *Proc. SPIE* **2014**, *9128*, 91280O-1-11.
62. Koerdt, M.; Kibben, S.; Hesselbach, J.; Brauner, C.; Herrmann, A. S.; Vollertsen, F.; Kroll, L. Fabrication and characterization of Bragg gratings in a graded-index perfluorinated polymer optical fiber. *Procedia Technol.* **2014**, *15*, 138- 146.
63. Sáez-Rodríguez, D.; Nielsen, K.; Bang, O.; Webb, D. J. Photosensitivity mechanism of undoped poly(methyl methacrylate) under UV radiation at 325 nm and its spatial resolution limit. *Opt. Lett.* **2014**, *39*, 3421-3424.
64. Sáez-Rodríguez, D.; K.Nielsen; O.Bang; D.J.Webb. Increase of the photosensitivity of undoped poly(methylmethacrylate) under UV radiation at 325 nm. *Proc. SPIE* **2014**, *9128*, 91280P-1-6.
65. Kowal, D.; Statkiewicz-Barabach, G.; Mergo, P.; Urbanczyk, W. Microstructured polymer optical fiber for long period gratings fabrication using an ultraviolet laser beam. *Optics Letters* **2014**, *39*, 2242-2245.
66. Hu, X.; Pun, C.-F. J.; Tam, H.-Y.; Mégret, P.; Caucheteur, C. Tilted Bragg gratings in step-index polymer optical fiber. *Opt. Lett.* **2014**, *39*, 6835-6838.
67. Hu, X.; Pun, C.-F. J.; Tam, H.-Y.; Mégret, P.; Caucheteur, C. Highly reflective Bragg gratings in slightly etched step-index polymer optical fiber. *Opt. Express* **2014**, *22*, 18807-18817.
68. Rajan, G.; Noor, M. Y. M.; Ambikairajah, E.; Peng, G. D. Inscription of multiple Bragg gratings in a single-mode polymer optical fiber using a single phase mask and its analysis. *IEEE Sens. J.* **2014**, *14*, 2384-2388.
69. Oliveira, R.; Marques, C. A. F.; Bilro, L.; Nogueira, R. N. Production and characterization of Bragg gratings in polymer optical fibers for sensors and optical communications. *Proc. SPIE* **2014**, *9157*, 915794-1-4.
70. Fasano, A.; Woyessa, G.; Stajanca, P.; Markos, C.; Stefani, A.; Nielsen, K.; Rasmussen, H. K.; Krebber, K.; Bang, O. Production and characterization of polycarbonate microstructured polymer optical fiber Bragg grating sensor. In *24th International Conference on Plastic Optical Fibers,* Nuremberg, Germany, 22-24 Sep. 2015.
71. Lacraz, A.; Polis, M.; Theodosiou, A.; Koutsides, C.; Kalli, K. Femtosecond laser inscribed Bragg gratings in low loss CYTOP polymer optical fiber. *IEEE Photon. Technol. Lett.* **2015**, *27*, 693-696.
72. Kalli, K.; Lacraz, A.; Polis, M.; Othonos, A. Femtosecond laser inscription of Bragg and complex gratings in coated and encapsulated silica and low-loss polymer optical fibers. *Proc. SPIE* **2015**, *9634*, 96343N-1-4.

73. Woyessa, G.; Fasano, A.; Stefani, A.; Markos, C.; Nielsen, K.; Rasmussen, H. K.; Bang, O. Humidity insensitive step-index polymer optical fibre Bragg grating sensors. *Proc. SPIE* **2015**, *9634*, 96342L-1-4.
74. Fasano, A.; Woyessa, G.; Stajanca, P.; Markos, C.; Stefani, A.; Nielsen, K.; Rasmussen, H. K.; Krebber, K.; Bang, O. Fabrication and characterization of polycarbonate microstructured polymer optical fibers for high-temperature-resistant fiber Bragg grating strain sensors. *Opt. Mater. Express* **2016**, *6*, 649-659.
75. Bundalo, I.-L.; Nielsen, K.; Bang, O. Angle dependent fiber Bragg grating inscription in microstructured polymer optical fibers. *Opt. Express* **2015**, *23*, 3699-3707.
76. Bundalo, I. L.; Nielsen, K.; Bang, O. Analysis of the angle dependency in inscription of the fiber Bragg gratings in the microstructured polymer optical fibers, In *2015 European Conference on Lasers and Electro-Optics*, Munich, 21 Jun. 2015; OSA.
77. Sáez-Rodríguez, D.; Nielsen, K.; Bang, O.; Webb, D. J. Time-dependent variation of fiber Bragg grating reflectivity in PMMA-based polymer optical fibers. *Opt. Lett.* **2015**, *40*, 1476-1479.
78. Oliveira, R.; Bilro, L.; Nogueira, R. Bragg gratings in a few mode microstructured polymer optical fiber in less than 30 seconds. *Opt. Express* **2015**, *23*, 10181-10187.
79. Chen, X.; Zhang, W.; Liu, C.; Hong, Y.; Webb, D. J. Enhancing the humidity response time of polymer optical fiber Bragg grating by using laser micromachining. *Opt. Express* **2015**, *23*, 25942-25949.
80. Kowal, D.; Statkiewicz-Barabach, G.; Mergo, P.; Urbanczyk, W. Inscription of long period gratings using an ultraviolet laser beam in the diffusion-doped microstructured polymer optical fiber. *Appl. Opt.* **2015**, *54*, 6327-6333.
81. Hu, X.; Kinet, D.; Chah, K.; Mégret, P.; Caucheteur, C. Bragg gratings inscription in step-index PMMA optical fiber by femtosecond laser pulses at 400 nm. *Proc. SPIE* **2016**, *9916*, 99161X-1-4.
82. Woyessa, G.; Fasano, A.; Stefani, A.; Markos, C.; Nielsen, K.; Rasmussen, H. K.; Bang, O. Single mode step-index polymer optical fiber for humidity insensitive high temperature fiber Bragg grating sensors. *Opt. Express* **2016**, *24*, 1253-1260.
83. Oliveira, R.; Bilro, L.; Marques, T. H. R.; Napierala, M.; Tenderenda, T.; Mergo, P.; Nasilowski, T.; Cordeiro, C. M. B.; Nogueira, R. Bragg gratings inscription in highly birefringent microstructured POFs. *IEEE Photon. Technol. Lett.* **2016**, *28*, 621-624.
84. Bhowmik, K.; Peng, G.-D.; Luo, Y.; Ambikairajah, E.; Lovric, V.; Walsh, W. R.; Rajan, G. Etching process related changes and effects on solid-core single-mode polymer optical fibre. *IEEE Photon. J.* **2016**, *8*, 2500109.

© 2017 by the authors. Submitted for possible open access publication under the terms and conditions of the Creative Commons Attribution (CC-BY) license (http://creativecommons.org/licenses/by/4.0/).
